# Thyroid hormone membrane receptor binding and transcriptional regulation in the sea urchin *Strongylocentrotus purpuratus*

**DOI:** 10.3389/fendo.2023.1195733

**Published:** 2023-05-26

**Authors:** Elias Taylor, Hannah Wynen, Andreas Heyland

**Affiliations:** Department of Integrative Biology, University of Guelph, Guelph, ON, Canada

**Keywords:** thyroid, nongenomic, genomic, transcriptome, sea urchin, echinoderm, thyroid hormone response element, skeletogenesis

## Abstract

Thyroid hormones (THs) are small amino acid derived signaling molecules with broad physiological and developmental functions in animals. Specifically, their function in metamorphic development, ion regulation, angiogenesis and many others have been studied in detail in mammals and some other vertebrates. Despite extensive reports showing pharmacological responses of invertebrate species to THs, little is known about TH signaling mechanisms outside of vertebrates. Previous work in sea urchins suggests that non-genomic mechanisms are activated by TH ligands. Here we show that several THs bind to sea urchin (*Strongylocentrotus purpuratus*) cell membrane extracts and are displaced by ligands of RGD-binding integrins. A transcriptional analysis across sea urchin developmental stages shows activation of genomic and non-genomic pathways in response to TH exposure, suggesting that both pathways are activated by THs in sea urchin embryos and larvae. We also provide evidence associating TH regulation of gene expression with TH response elements in the genome. In ontogeny, we found more differentially expressed genes in older larvae compared to gastrula stages. In contrast to gastrula stages, the acceleration of skeletogenesis by thyroxine in older larvae is not fully inhibited by competitive ligands or inhibitors of the integrin membrane receptor pathway, suggesting that THs likely activate multiple pathways. Our data confirms a signaling function of THs in sea urchin development and suggests that both genomic and non-genomic mechanisms play a role, with genomic signaling being more prominent during later stages of larval development.

## 1 Introduction

Thyroid hormones are critical for development and metamorphosis of chordate deuterostomes, regulating a diversity of physiological systems (e.g. corticogenesis, angiogenesis, skeletogenesis, apoptosis, and cell cycle). Evidence suggests that THs may also play an essential role in non-chordate metazoans, however, the mechanism and function of THs in these groups remains unclear (reviewed in 1 and 2). THs have been shown to accelerate development to metamorphosis in echinoderms, including sea urchins, sand dollars, and sea stars (3–9) as well inducing metamorphosis in mollusks (10, 11) and potentially regulating skeletogenesis in cnidarians and echinoderms (12, 13).

In vertebrates, two major TH signaling mechanisms have been shown to act, genomic and non-genomic. While the mechanisms and functions of non-genomic signaling remains relatively poorly understood, the canonical genomic pathway has been studied extensively in vertebrates (14, 15). When acting genomically, THs regulate genes in diverse tissues performing a wide array of physiological functions. TH regulation of gene expression is crucial for development of neural structures, regulation of metabolism, metamorphosis, apoptosis, immune function, and more (16–22). THs, primarily T4, are synthesized in the thyroid gland; a chordate innovation (23). T4 and T3 are transported through the cell membrane where T4 is deiodinated into T3. T3 is translocated into the nucleus, binding to the TH nuclear receptor (TR) in a complex with the retinoid X receptor (RXR) and other associated proteins. This complex binds to TH response elements in the genome and promotes or inhibits gene expression. Thyroid response elements comprise a pair of repeated motifs, one of which is bound by RXR and one by TR. The spacing and sequence of the repeated motifs specifies the nuclear hormone receptor bound (24). As an example, the canonical TH response element sequence is 5’-AGGTCA-3’, with 4-nucleotide spacing between repeated elements, referred to as Direct Repeat 4 (DR4). In contrast, the vitamin D receptor (VDR) and retinoic acid receptor (RAR) are hypothesized to prefer a 3- or 5-nucleotide spacing, respectively. However, thyroid hormone receptors can diverge from the spacing or sequence requirements, and the nucleotide spacers may be non-random—potentially resulting in greater specificity for THR over VDR or RAR (25, 26).

Information on nongenomic actions of THs have emerged in the context of neurogenesis, neural signaling, vasculogenesis, metabolism, actin structure, and more (14, 27–32). THs can act non-genomically by binding to an integrin membrane receptor in chordates and potentially echinoderms (13, 14). In chordates, T4 and T3, but primarily T4, bind to the RGD-binding pocket on integrin αVβ3. This causes a conformational change in the cytoplasmic tail of the integrin, recruiting protein kinase C (PKC) and associated proteins (e.g. Phospholipase C) before triggering a mitogen-activated protein kinase (MAPK) cascade via the MAPK kinase MEK 1/2 and the MAPK ERK 1/2 (reviewed in 14). This MAPK cascade phosphorylates transcription factors and enzymes, having differential effects depending on cell type. A number of additional non-genomic pathways wherein THs bind to an extranuclear TR have also been described (14, 33–35; N. P. 36) and these pathways may also result in regulation of gene transcription (14, 30). There is evidence that some of these pathways may be conserved among bilaterians (37).

Previously, we have shown that THs can accelerate skeletogenesis in sea urchin gastrulae, and in developing metamorphic structures of late-stage pluteus larvae (3, 8, 9, 13). Furthermore, we showed that TH is in part responsible for reduced larval arm length via programmed cell death in late-stage larvae which have begun developing metamorphic structures (post-ingression larvae), but not larvae which had not yet begun development of metamorphic structures (pre-ingression larvae; 38).

To investigate the mechanisms of TH action in echinoderms, we have conducted binding assays between THs and membrane protein extract from sea urchin gastrulae, hypothesizing that THs would bind to membrane proteins and be displaced by ligands of RGD-binding integrins. Furthermore, we analyzed the transcriptional response of sea urchin embryos and larvae in response to TH treatments (T3 and T4) and conducted a TH response element enrichment analysis on differentially regulated genes and genes with nearby detected TRE motifs.

## 2 Materials and methods

### 2.1 Animal care

Adult *S. purpuratus* urchins were obtained from Monterey, CA and transported to the Hagen Aqualab at the University of Guelph, ON, where they were kept in tanks of filtered artificial seawater (FASW). They were fed kelp (*Macrocystis pyrifera* and *Kombu* spp.) every 2-3 days and maintained at a temperature of 12-14°C and a salinity of 31 g/L. The urchins were spawned using an injection of 0.5-2 mL of 0.5 M KCl. Sperm was collected using a pipette, while females were inverted over a beaker of FASW to collect eggs, which were then filtered to remove debris and washed twice with filtered artificial seawater. The sperm was diluted in 1 mL of FASW and added slowly to the beaker of eggs until fertilization success, determined by the presence of a fertilization envelope, reached over 90%. Fertilized eggs were washed once more to remove excess sperm and allowed to develop at 12°C in a 1L beaker until hatching. Hatched embryos were transferred to 2L beakers at a density of 1 larvae/mL and maintained at 12-14°C and a salinity of 31-33 g/L. As the larvae aged, density was reduced to 0.15 larvae/mL by the eight-arm stage, splitting cultures as necessary. The larval cultures were constantly stirred and kept on a 12:12 light cycle, with cleaning and water replacement performed three times weekly. At each cleaning, the cultures were fed *Rhodomonas* sp. and *Dunaliella salina*. with a total density of 5000 cells/mL.

### 2.2 Skeletogenic assays

Skeletogenic assays were conducted as described in Taylor and Heyland (13), modified for rudiment development instead of gastrulation. Larvae were collected after rudiment development had begun, but prior to skeletogenesis in the rudiment (soft tissue stage iii-iv in 39). Larvae were kept in 24 well plates at a density of 20 embryos/mL for gastrulae, or 1 larvae/mL for pluteus larvae. The plates were kept in an incubator at 12°C on a shaker table. Water was changed and larvae were fed 6,000 c/mL Rhodomonas sp. every 2 days.

Over the course of 5 days, larvae were exposed to a vehicle control (0.005% DMSO), or to thyroid hormone analogs rT3, T4, T3, T2, Triac, and Tetrac at 10^-7^ M, a competitive inhibitor of the integrin membrane receptor binding site, RGD peptide (10^-7^ M) or an inhibitor of ERK1/2 activity, PD98059 (5 x 10^-7^ M). Larvae were also exposed to a combination of RGD and T4, or PD98059 and T4, to determine if there was an inhibitory effect on the action of T4.

We scored the presence of skeletal spicules in the rudiment and determined the proportion of individuals which had developed skeleton in the rudiment. Skeletal structures were detected by polarized light microscopy on a Nikon Ti2 compound inverted microscope. Rate of skeletogenesis was calculated by the average daily proportion of larvae with new spicules and compared to the control with a two-tailed t-test.

### 2.3 Binding assays

Membrane protein extracts were obtained from *S. purpuratus* gastrulae. A membrane protein extract from the breast cancer cell line MDA-MB-231, an epithelial cell line known to express integrin αVβ3, was used as a positive control (provided by Dr. M. Coppolino at the University of Guelph). To collect gastrula membrane proteins, 36-hr old larvae were gently concentrated by centrifugation (3000 rpm) before being collected by pipette. 30 µL of gastrulae per sample were processed with a Mem-PER™ Plus kit (Thermo Scientific 89842). Final membrane-enriched protein yield was 2.01 mg – 2.96 mg as measured by NanoDrop A280 and standard BCA protein assay respectively. Protein extracts were stored at -80°CC.

Fluorescently labeled thyroxine (RHT4) was synthesized as described in 13. For the saturation assay, 18 tubes were prepared and 940 µL of membrane extraction buffer was added to each tube. RHT4 was premixed with 2% BSA and then added to the membrane extraction buffer in concentrations starting at 2.7 x 10^-4^ M and serially diluted 3-fold, 17 times, to a minimum concentration of 2.1 x 10^-12^ M. Then, 60 µL of the membrane protein extract was added to the membrane extraction buffer and allowed to equilibrate for one hour. Subsequently, the mixture was centrifuged for 1 minute. The supernatant was removed, and 1 mL of membrane extraction buffer was added to the remaining pellet, which was then vortexed and centrifuged for 1 minute. The supernatant was once again removed, and 180 µL of membrane extraction buffer was added to the pellet. Fluorescent intensity was measured on a POLARstar Omega plate reader at excitation/emission 544/590 nm. This assay was repeated three times (n=3).

For the fluorescence anisotropy competitive binding assays, 50 µL of membrane protein extract, RHT4 at 10^-7^ M, and enough PBS buffer to total 200 µL was added to 18 wells in a 96-well plate. The competitive ligand was then added (T4, T3, rT3, RGD, T2, Tetrac, or Triac). A serial dilution of this ligand of concentrations starting at 2.7 x 10^-4^ M and serially diluted 3-fold, 17 times, to a minimum concentration of 2.1 x 10^-12^ M was added. The plate was allowed to equilibrate for one hour. Fluorescent polarization was measured on a POLARstar Omega plate reader at excitation/emission 520/590 nm. This assay was repeated three times for each competitive ligand (n=3).

Ligand binding curves and Ki/Kd (inhibition and dissociation constants) were calculated with GraphPad Prism 9.5.1 for Windows using a nonlinear fit. Saturation binding and competitive binding were analyzed with the “One Site – Total Binding” and “One Site – Fit Ki” modules respectively.

### 2.4 Transcriptome collection and DEG analysis

We conducted an RNAseq experiment for a total of 18 samples with two treatment groups, T3-exposed and T4-exposed, and at three stages of larval development: 48h (gastrulation), 23 days post-fertilization (pre-rudiment formation), and 27 days post-fertilization (developing juvenile rudiment). RNA extractions were conducted using the Direct-zol ^®^ RNA MiniPrep Kit (Zymo Research). The RNA samples were examined using a Nanodrop 8000 Spectrophotometer. Library preparation and sequencing of samples was performed by The Hospital for Sick Children’s Center for Applied Genomics (Toronto, ON) on an Illumina HiSeq2500 System.

Transcripts were trimmed using Trim Galore! (v0.6.4; 40) and CutAdapt (v2.6; 41) to remove adaptors and poor-quality reads. Reads with a Phred quality score lower than 5 or a length shorter than 36 bases were discarded. Otherwise, default settings were used. Quality metrics and read counts can be found in **Supplemental S4**. Trimmed reads were aligned to the *S. purpuratus* genome (v. 5.0; 42) and quantified using the align_and_estimate_abundace.pl script from Trinity (v.2.8.6; 43) with RSEM (v.1.3.1; 44) as the chosen method of quantification. The gene to transcript mapping file was generated from the *S. purpuratus* genome files, with each mRNA locus considered to be a gene object.

DESeq2 (v.1.30.0; 45) was used to normalize read counts as trimmed mean of M values (TMM) for downstream analysis. DESeq2 was also used to conduct pairwise comparisons of each treatment group at each developmental stage to the control group at the same developmental stage.

In addition to automated GO annotation, genes in the *S. purpuratus* genome (v. 5.0; 42) were also manually annotated by functional group. Manual annotation was necessary to update previous functional annotations for the *S. purpuratus* 5.0 genome, and to incorporate recent work. In order of mention: The *spicule proteome* was updated from Mann et al. (46). *PMC-expressed* genes were obtained from Rafiq et al. (47). The *skeletogenic GRN* list was compiled from the Davidson Lab Gene Regulatory Network model hosted on BioTapestry. *TH Transport*, *Signaling*, *Synthesis*, *Sulfotransferase*, *Nuclear hormone receptor*, and *apoptosis-related* genes were manually annotated using BLAST, from chordate orthologs, as well as from annotations available on Echinobase. The *integrin adhesome* was compiled using BLAST on the consensus adhesome from (48) as well as searching for similarly annotated genes on Echinobase. The *cadherin* **“** *cadhesome* **”** was sourced from (49) in a similar fashion. Lists of *integrins* and *cadherins* were obtained by BLAST-ing known chordate integrins and cadherins against the *S. purpuratus* genome. *Neuronal* genes were manually annotated by BLAST-ing chordate synthesis, transport, and receptor genes against the *S. purpuratus* genome as well as from 50–55; and 56. Immune gene list was sourced from Rast et al. (57). R Scripts used are available in **Supplement S3**.

### 2.5 UMAP and heatmap clustering

The heatmaps were created and clustered using the *ComplexHeatmaps* R package (58) with the default Euclidean distance and partitioned by k-means. UMAP dimension reduction visualization was performed using the *umap* package (59) implementing the UMAP algorithm (described in 60).

### 2.6 Thyroid hormone response element analysis

We analyzed the average number of thyroid hormone response elements near DEGs and average log-fold-change of DEGs near TH response elements. The “Find Motif” function in Geneious Prime 2022 (2022.0.2) was used to search for the canonical TH and RXR response element sites in the *S. purpuratus* genome (v. 5.0; 42). The search sequence used for the most permissive search allowing for 0-6 bases spacing the repeated TRE halfsites was [AG]-[G]-G-[AT]-C-A-N(0,6)-[AG]-[G]-G-[AT]-C-A with two allowed mismatches. For halfsites alone, the search used was [AG]-G-G-N-C-A with no permitted mismatches. For the canonical DR4 site, the search used was [AG]-[G]-G-N-C-A-N-N-N-N-[AG]-[G]-G-N-C-A. Evidence suggests that these motifs are conserved between protostomes and deuterostomes (61, 62). Sequence logos were graphed with seqLogo (63).

Motif sites were exported as a CSV and a custom python script was used to tally the number of sites and their distance to every gene in the genome. The average log-fold change of each gene with a large number of TREs within 500bp upstream of initiation was compared to genes with no nearby TREs. The number of TRE sites was chosen such that at least 50 DEGs were included in the sample: 10 TREs for the Halfsite and DR0-6 analysis, and 8 TREs for the DR4 analysis, owing to the lower number of detected DR4 sites. The number of DEGs within 500 bp upstream of initiation of DEGs was also compared to non-DEGs. All statistical comparisons were two-tailed T tests.

## 3 Results

### 3.1 Thyroid hormones bind to membrane protein extract and can be displaced by RGD peptide

**Figure 1:**
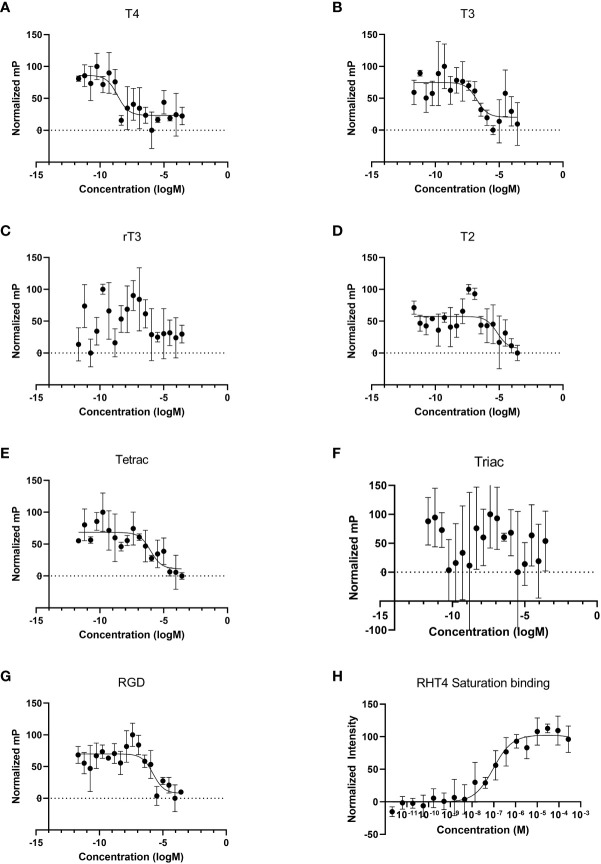
Thyroid hormones bind to membrane protein extracts from *Strongylocentrotus purpuratus* gastrulae and can be displaced by integrin ligands. **(A–G)** In fluorescence anisotropy competitive binding assays, T4, T3, T2, Tetrac, and RGD peptide bind to membrane protein extract. Normalized mP: Millipolarization normalized on a scale of 0-100. We were not able to detect binding of rT3. Out of the tested ligands, T4 showed the highest affinity for membrane proteins, with a calculated Ki of approximately 1.1 x 10^-9^ M. **(H)** In a saturation binding assay, thyroxine labeled with rhodamine (RHT4) binds to membrane protein extract with a Kd of approximately 9.5 x 10^-8^ M, roughly two orders of magnitude lower in affinity when compared to unlabeled T4.

We performed competitive binding assays using fluorescence anisotropy on membrane protein extract from sea urchin gastrulae (**Figures 1A–G**). The thyroid hormones, T4, T3, and T2, as well as Tetrac (a TH analogue) and RGD peptide (a ligand of RGD-binding integrins) were found to specifically bind to membrane proteins and displaced rhodamine-labeled T4 (RHT4). **Table 1** summarized the binding affinities extracted from the binding curves in **Figure 1**. Out of the tested ligands, T4 showed the highest affinity for membrane proteins (Ki, 95% CI = 3.8 x 10^-10^ – 3.7 x 10^-9^; **Table 1**.), followed by T3 (Ki, 95% CI = 1.9 x 10^-8^ – 3.6 x 10^-7^). A saturation binding assay was performed with RHT4 alone to validate its use as a fluorescent ligand for the competitive binding assay and it was found to have a similar affinity to T3 (Kd, 95% CI = 4.5 x 10^-8^ – 2.0 x 10^-7^).

**Table 1:** Binding constants of thyroid hormones and integrin ligands with membrane protein extracts from *Strongylocentrotus purpuratus* gastrulae, as calculated from **Figure 1**.

Ligand	Ki (95% CI)
Thyroxine (**T4**)	1.1 x 10^-9^ (3.8 x 10^-10^ – 3.7 x 10^-9^)
3,5,3’-Triiodo-l-thyronine (**T3**)	8.8 x 10^-8^ (1.9 x 10^-8^ – 3.6 x 10^-7^)
3,3′,5′-Triiodo-thyronine (**rT3**)	–
3,5-Diiodo-l-thyronine (**T2**)	3.4 x 10^-6^ (4.0 x 10^-7^ – 5.6 x 10^-5^)
Tetraiodothyroacetic Acid (**Tetrac**)	4.5 x 10^-7^ (7.7 x 10^-8^ – 5.4 x 10^-6^)
Triiodothyroacetic acid (**Triac**)	–
Arg-Gly-Asp peptide (**RGD**)	8.2 x 10^-7^ (2.9 x 10^-7^ – 2.5 x 10^-6^)
Rhodamine-conjugated T4 (**RHT4**)	9.5 x 10^-8 *^ (4.5 x 10^-8^ – 2.0 x 10^-7^)

* Kd.

### 3.2 Thyroid hormones induce skeletogenesis in sea urchin rudiment

**Figure 2:**
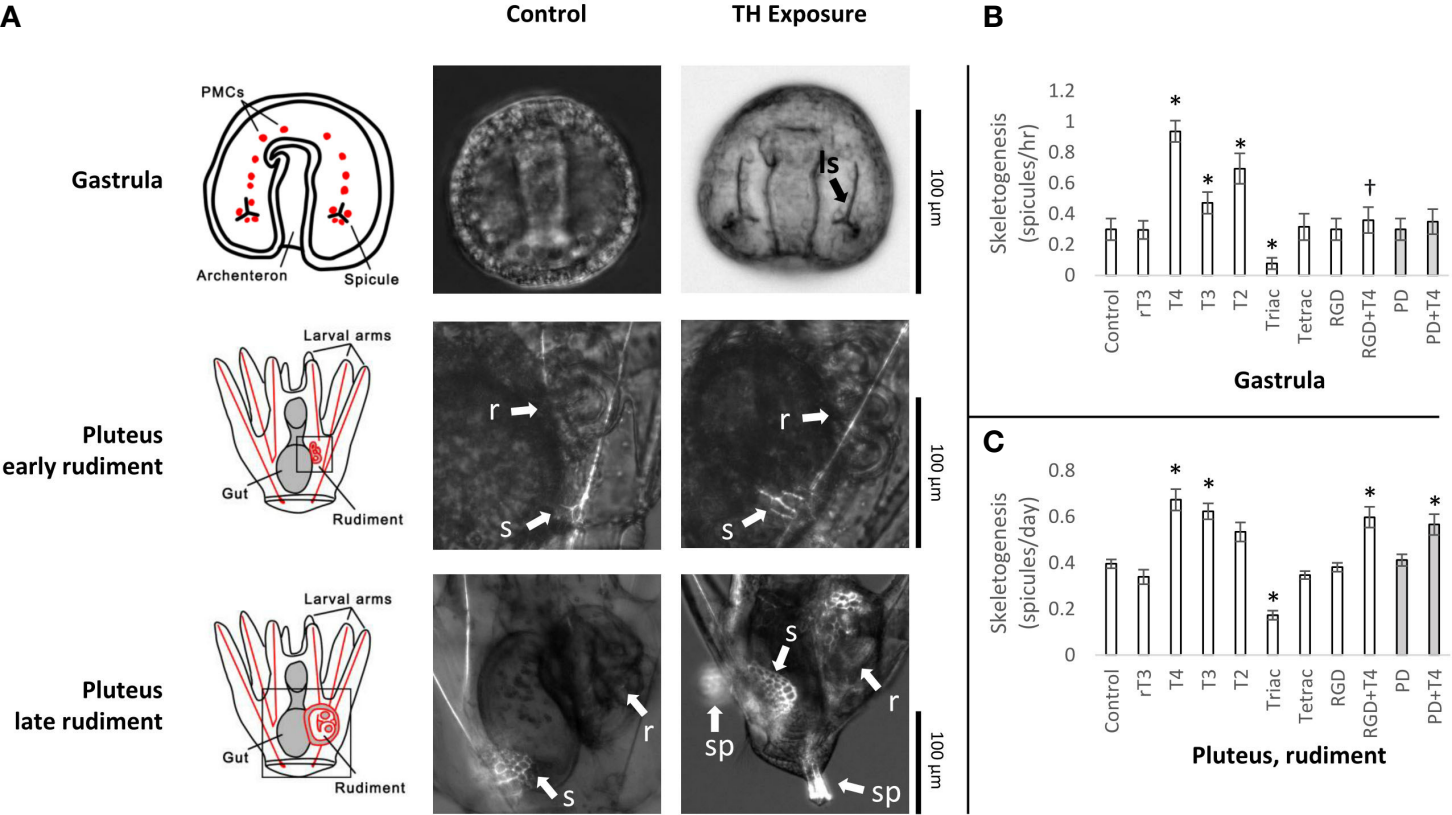
Thyroid hormone exposure accelerates skeletogenesis in *Strongylocentrotus purpuratus* larvae. **(A)** Representative images of TH exposure effects on skeletogenesis in the gastrula and late-stage larvae. In gastrulae, TH exposure accelerates initiation of skeletogenesis with initial spicules for larval skeleton appearing several hours earlier compared to the control (no TH). In the rudiment, THs accelerate development of skeletal features, including tube feet, spines, and test. ls: Larval skeleton, r: Rudiment, s: Skeleton, sp: Juvenile spines. **(B)** THs accelerate skeletogenesis in Gastrula in a MAPK-dependent manner. Data reproduced from 13. All THs and RGD peptide are shown at a concentration of 10^-7^M, PD98059 at 5 x 10^-6^M. * indicates a rate of skeletogenesis statistically different from the control (Binary logistic regression with Bonferroni corrected p-values). THs, including T4, T3, and T2, accelerate skeletogenesis.†: While we have displayed here all THs at 10^-7^M, a higher concentration of T4 is able to outcompete the inhibitory effect of RGD peptide, as discussed in 13. **(C)** THs accelerate skeletogenesis in late-stage larvae in a partially MAPK-dependent manner, including T3 and T4 (One-way ANOVA with Bonferroni-corrected t tests; F(10,121) = [20.88], p = 4.96e-22). The effect of T2 was not significant after correcting for multiple comparisons (adjusted p = 0.063). Unlike in gastrulae, inhibiting MAPK with PD98059 is insufficient to fully prevent the effect of T4 on skeletogenesis. * indicates a rate of skeletogenesis statistically different from the control (Bonferroni-corrected t-test, p<0.05).

Thyroid hormones (T4, T3, and T2) at 10^-7^M induced skeletogenesis in sea urchin larvae in the juvenile rudiment, as measured by the rate of initial skeletal spicule formation (1.70x, 1.57x, 1.35x respectively; t-test, p<0.05; **Figures 2A, C**). The TH analog, Triac, was found to inhibit skeletogenesis (2.28x reduction; t-test, p<0.05; **Figure 2C**). Effects of THs on skeletogenesis in the rudiment were lessened by RGD peptide and PD98059, but not to a statistically significant degree. This contrasts with skeletogenesis in gastrulae which was significantly inhibited by RGD peptide and PD98059 (13, **Figure 2B**). As well, we observed a greater degree of skeletogenesis overall in older larvae, with T4-exposed samples displaying the most advanced degree of skeletal development (**Figure 2A**). Although we did not quantitatively measure this, T4-exposed samples displayed complete juvenile spines, denser skeletal growth proximal to the gut, and a more advanced stage of skeleton in the rudiment (representative images in **Figure 2A**).

### 3.3 Patterns of gene expression on exposure to the thyroid hormones T3 and T4

**Figure 3:**
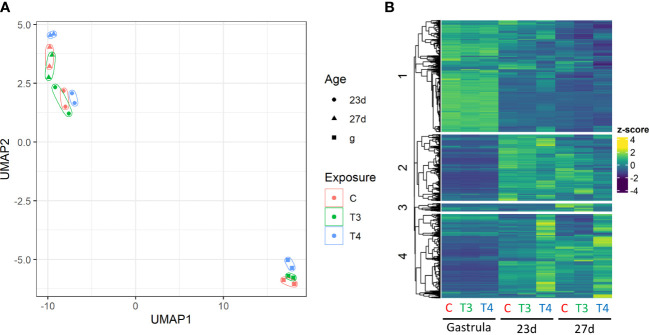
Summary of transcriptome data structure. Gene expression clusters by age and hormone treatment T4-exposed samples are dissimilar from control and T3-exposed samples within each age group. **(A)** UMAP analysis of individual replicates reveals clustering primarily by age, with highly divergent gene expression patterns between the gastrulae and older larvae groups. Control and T3-exposed groups cluster more tightly together than with T4-exposed groups. **(B)** Heatmap of gene expression with genes separated into 4 clusters by k-means using the ComplexHeatmap R package. Gene counts are normalized to z-scores. The heatmap reveals that genes are differentially regulated in gastrulae and older larvae (9613 DEGs between 27-day old and gastrulae control groups), as well as in T4-exposed groups relative to control and T3-exposed groups in older larvae (1730 DEGs in T4 27d relative to control).

When compared to the control, 2816 genes were differentially regulated in thyroid hormone-exposed groups (T3, T4; **Figure 3**). UMAP analysis revealed clustering of gene expression was structured primarily by age, and secondarily by hormone exposure. Within each age group, expression of the control and T3-exposed groups clustered more closely with each other than with the T4-exposed group (**Figure 3A**). Of genes that were differentially regulated in both 23 day and 27 day old samples (685/690), 97.8% were regulated to a greater degree by T4. In contrast, 50.9% of DEGs in gastrulae (79/155) were regulated to a greater degree by T4 (**Figure 3B**).

The greatest number of DEGs (2441 total) were found in T4-exposed older larvae (23 and 27 days post-fertilization; logfoldchange and DESeq2-derived p-values of DEGs listed in **Supplement S1**). Genes can broadly be separated into those expressed in gastrulae, and those expressed in older larvae, as well as by those regulated and not regulated by T4. In gastrulae, only 155 DEGs were found to be regulated by T4, while in 23d and 27d groups 1597 and 1730 DEGs were found respectively, over a 10-fold increase in the number of regulated genes. As well, we noted that more DEGs were upregulated by T4 and not downregulated (58%). This trend was reversed for T3, with more DEGs being downregulated (43%).

### 3.4 Thyroid hormones regulate gene functional groups

**Figure 4:**
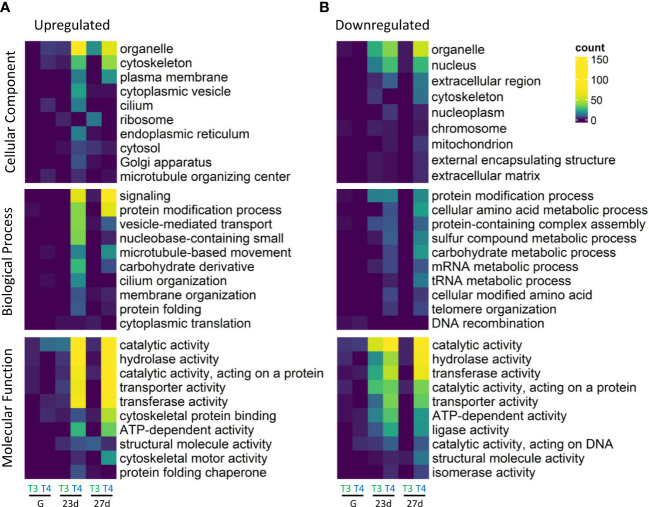
Summary of top ten significantly enriched GO slim categories of upregulated and downregulated genes. Gene ontology (GO) enrichment was determined by Fisher’s exact test in T3 and T4-exposed gastrulae (G), 23-day old larvae (23d), and 27-day old larvae (27d). More GO categories were enriched in the upregulated subset of DEGs. The most differentially enriched groups between **(A)** upregulated and **(B)** downregulated DEGs in each major GO category were cytoskeleton, and plasma membrane (CC), signaling, vesicle-mediated transport, and microtubule-based movement (BP), and cytoskeletal protein binding and cytoskeletal motor activity (MF). Cells are coloured and GO slim categories are sorted by number of DEGs assigned to each GO slim category. Every displayed GO slim category was significantly enriched in at least one sample. Fisher’s exact test was used to determine significantly enriched GO categories relative to control groups. The top ten groups sorted by lowest p values (<0.05) are displayed for each major GO category (Cellular Component, Biological Process, and Molecular Function). GO annotations were sourced from Ensembl Metazoa and mapped to GO slim annotations.

GO annotation categories were enriched in the thyroid hormone-exposed groups (p <0.05, Fisher’s exact test; top ten categories displayed in **Figure 4**). Overall, the most enriched GOSlim group in each major GO category was organelle (Cellular component; CC), Signaling (Biological Process; BP), and catalytic activity (Molecular Function; MF). We saw high levels of enrichment of protein-modification process (BP), hydrolase activity, transporter activity, and transferase activity (MF) in the TH exposed groups.

The most enriched groups in the upregulated as compared to downregulated DEGs in each major GO category were cytoskeleton, and plasma membrane (CC), signaling, vesicle-mediated transport, and microtubule-based movement (BP), and cytoskeletal protein binding and cytoskeletal motor activity (MF). The most enriched groups in downregulated as compared to upregulated DEGs were nucleus and extracellular region (CC), several categories of metabolic processes including sulfur, carbohydrate, mRNA, and tRNA (BP), and ligase activity (MF).

**Figure 5:**
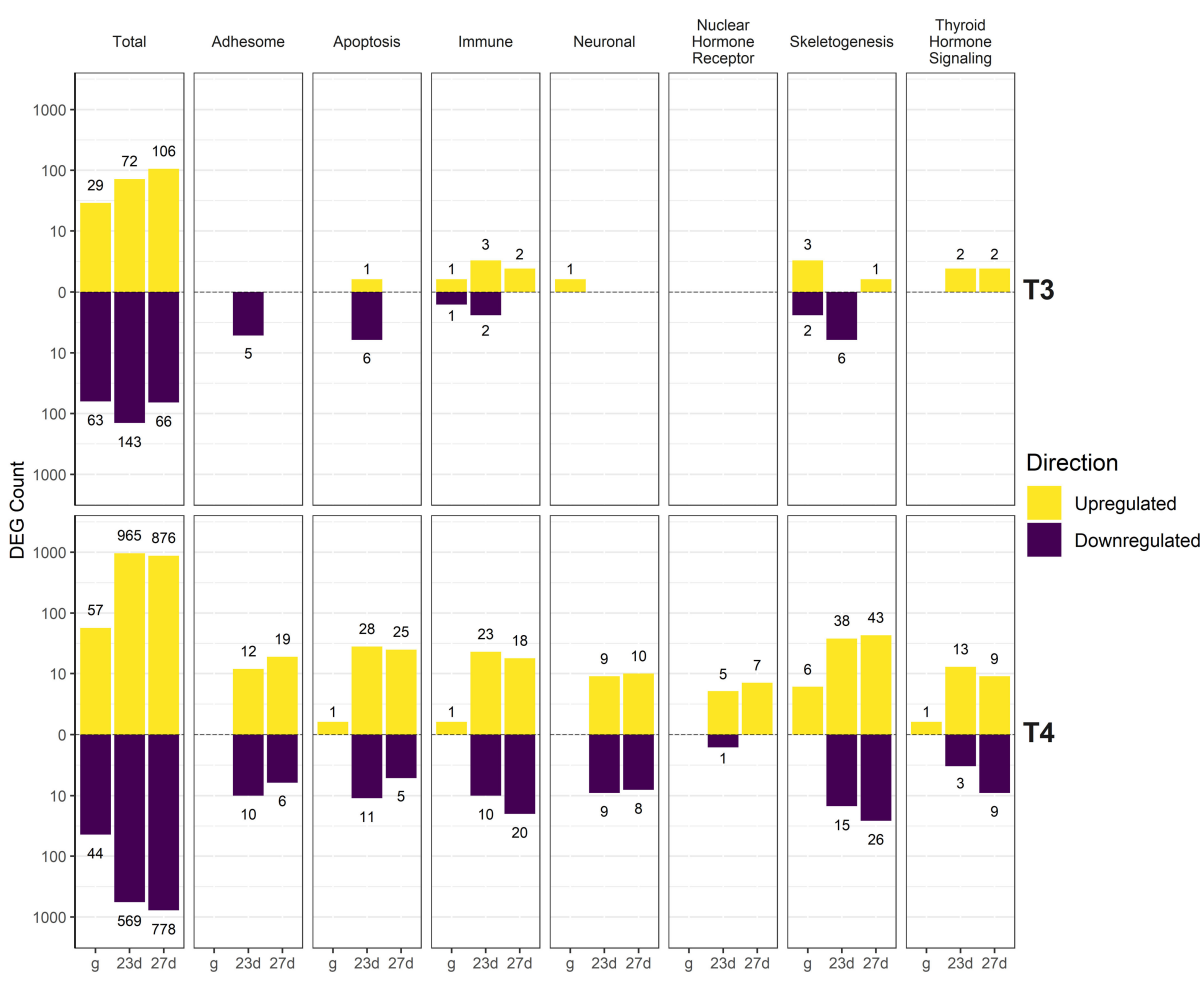
Summary of upregulated and downregulated genes by manually annotated functional group. DEGs were determined using DESeq2 comparisons between TH-exposed groups and the control group of the same age. T4 resulted in regulation of more genes than T3, with dramatically more DEGs in older larvae compared to gastrulae. More DEGs are upregulated than downregulated. Individual categories are explored in more detail in **Figures 6**–**12**.

**Figure 6:**
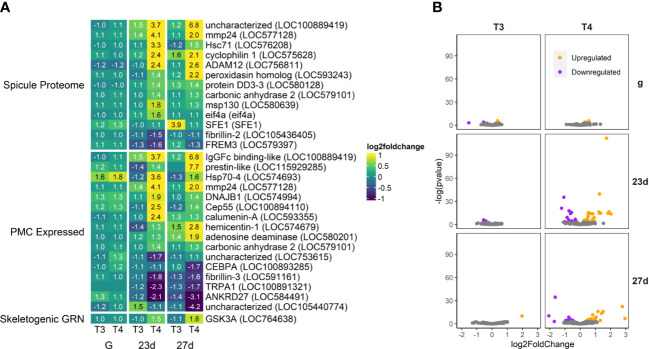
T4 regulates gene expression of skeletogenesis-related genes in older larvae. **(A)** Heatmap of top 30 skeletogenesis-related genes, sorted by p-value (low to high, determined by DESeq2) and clustered by expression pattern. Colours are scaled to log2(foldchange) and capped at 2-fold, while numbered cells display foldchange. Spicule proteome was updated from 46. PMC-expressed genes were obtained from 47. The Skeletogenic GRN list was compiled from the Davidson Lab Gene Regulatory Network model hosted on BioTapestry. **(B)** Volcano plots of all skeletogenesis related genes.

**Figure 7:**
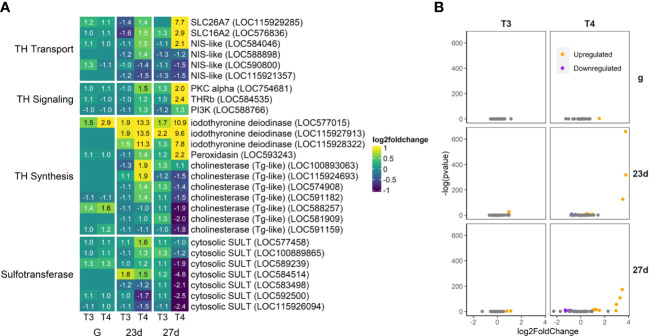
T4 regulates gene expression of thyroid hormone-related genes in older larvae. Deiodinases and some putative TH transporters are strongly upregulated. Tg-like cholinesterases (>40% identity to Tg with >4% tyrosine content) and sulfotransferases are generally downregulated in 27-day-old post-rudiment development larvae (27d). **(A)** Heatmap of top 30 TH-related genes, sorted by p-value (low to high, determined by DESeq2) and clustered by expression pattern. Colours are scaled to log2(foldchange) and capped at 2-fold, while numbered cells display foldchange. Genes were compiled from annotations in the *S. purpuratus* 5.0 genome release and verified with manual BLAST searches against chordate genomes. **(B)** Volcano plots of all TH-related genes.

**Figure 8:**
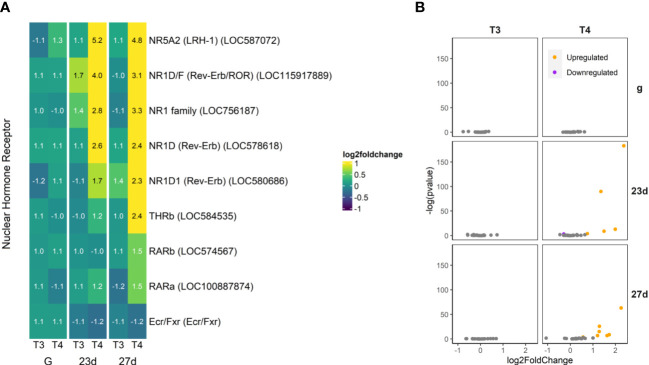
T4 regulates gene expression of nuclear hormone receptors in older larvae. Notably, all the upregulated nuclear receptors excepting NR5A2 are in the NR1 family. The putative nuclear TH receptor is among those genes found to be upregulated in larvae with rudiments (27d), but not in younger larvae without rudiments (23d). **(A)** Heatmap of top 30 nuclear hormone receptor genes, sorted by p-value (low to high, determined by DESeq2) and clustered by expression pattern. Colours are scaled to log2(foldchange) and capped at 2-fold, while numbered cells display foldchange. Genes were compiled from annotations in the *S. purpuratus* 5.0 genome release and verified with manual BLAST searches against chordate genomes. **(B)** Volcano plots of all nuclear hormone receptor genes.

**Figure 9:**
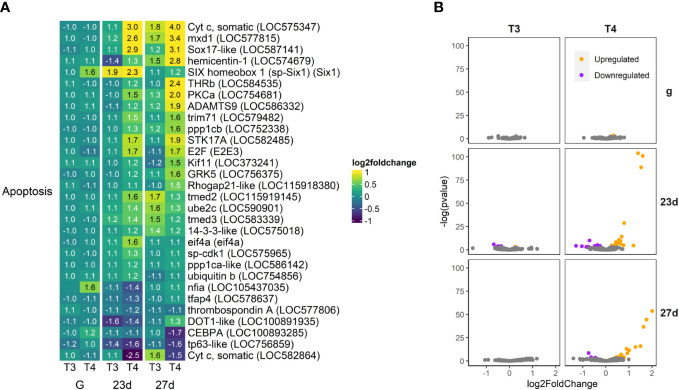
T4 regulates gene expression of apoptosis-related genes in older larvae. The most highly upregulated and downregulated genes are putative orthologs of cytochrome C (Cyt C), an inhibitor of apoptosis. **(A)** Heatmap of top 30 apoptosis-related genes, sorted by p-value (low to high, determined by DESeq2) and clustered by expression pattern. Colours are scaled to log2(foldchange) and capped at 2-fold, while numbered cells display foldchange. Apoptosis-related genes were compiled from the Reactome pathway database and matched to S. purpuratus via annotations available on Echinobase or by best BLAST match to the S.p. 5.0 genome. **(B)** Volcano plots of all apoptosis related genes.

**Figure 10:**
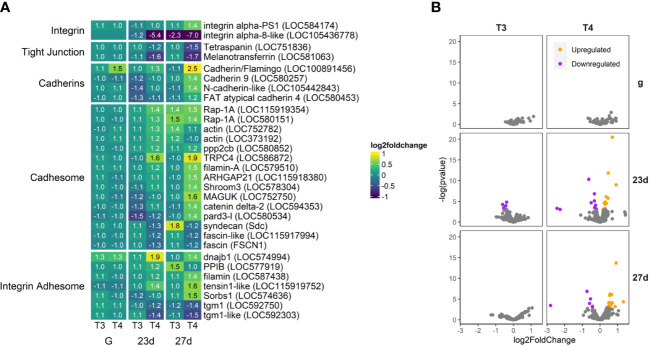
T4 regulates gene expression of adhesome-related genes in older larvae, however not to the extent of other categories we examine. Integrin alpha-8-like is dramatically downregulated, while integrin alpha-PS1 and several cadherins are moderately upregulated, along with elements of the Cadhesome and Adhesome. Transglutaminases are also notably downregulated in older larvae. **(A)** Heatmap of top 30 adhesome-related genes, sorted by p-value (low to high, determined by DESeq2) and clustered by expression pattern. Colours are scaled to log2(foldchange) and capped at 2-fold, while numbered cells display foldchange. Genes were compiled from annotations in the *S. purpuratus* 5.0 genome release and verified with manual BLAST searches against chordate genomes. **(B)** Volcano plots of all adhesome related genes.

**Figure 11:**
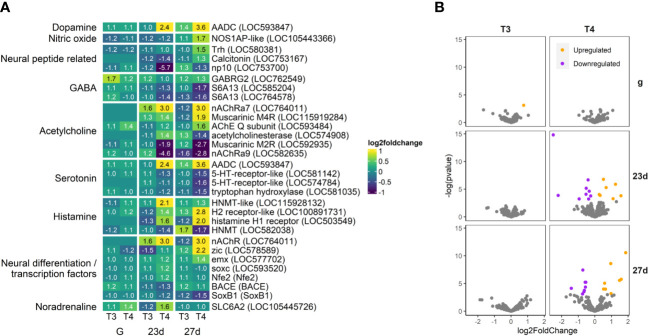
T4 regulates gene expression of neuronal genes in older larvae. Aromatic L-amino acid decarboxylase (AADC), a key component of dopamine and serotonin synthesis, is dramatically upregulated by T4 in older larvae. As well, histamine receptors and several acetylcholine receptors are also upregulated. Several serotonin and acetylcholine receptors are downregulated. **(A)** Heatmap of top 30 neuronal genes, sorted by p-value (low to high, determined by DESeq2) and clustered by expression pattern. Colours are scaled to log2(foldchange) and capped at 2-fold, while numbered cells display foldchange. Genes were compiled from annotations in the *S. purpuratus* 5.0 genome release and verified with manual BLAST searches against chordate genomes. **(B)** Volcano plots of all neuronal genes.

**Figure 12:**
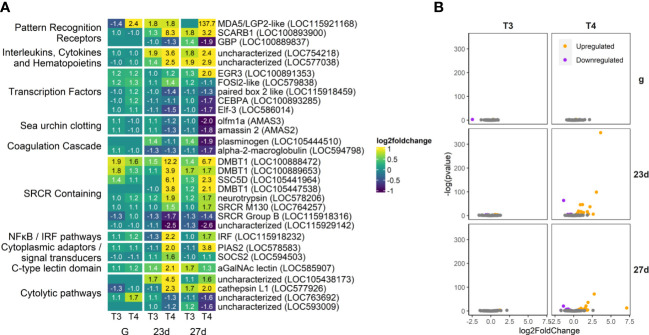
Some immune genes are strongly upregulated in our T4-exposed groups (5.7% of total annotated genes), including a variety of SRCR-domain containing proteins. Several clotting and coagulation-related genes were strongly downregulated by T4. **(A)** Heatmap of top 30 immune genes, sorted by p-value and clustered by expression pattern. Colours are scaled to log2(foldchange) and capped at 2-fold, while numbered cells display foldchange. Immune gene list was compiled and updated from 57. **(B)** Volcano plots of all immune genes.

Genes were also manually annotated by physiological function (summarized in **Figure 5**, full list of manually annotated genes in **Supplement S2**). DEGs were found in Skeletogenesis (**Figure 6**), TH Signaling (**Figure 7**), Nuclear Hormone Receptor (**Figure 8**), Apoptosis (**Figure 9**), Adhesome/Cadhesome (**Figure 10**), Neuronal (**Figure 11**), and Immune (**Figure 12**) functional groups. A high percentage of TH signaling-related and nuclear hormone receptor genes were differentially regulated (22.9% and 25.8% respectively, 22 and 8 total DEGs). 12.8% of manually annotated skeletogenesis-related genes were differentially regulated. This is higher than the percentage of annotated Apoptosis, Adhesome, Neuronal, and Immune genes regulated (9.2%, 9.9%, 4.1%, and 5.7% respectively). We found the highest absolute number of skeletogenesis-related DEGs (69 total genes).

#### 3.4.1 Skeletogenesis

Thyroxine (T4) regulates genes known to be expressed in skeletogenic mesenchyme cells in sea urchins. A total of 112 skeletogenesis-related DEGs were found. Effector genes (including spicule matrix proteins, matrix metalloproteases, and proteins associated with calcium carbonate production) involved with skeletogenesis were regulated by T4, but only a single high-level DEG controlling skeletogenic cell fate was detected (**Figure 6**; **Supplement S1**, **S2**).

Out of genes expressed in the spicule proteome (46 – the proteins involved in formation of the initial skeleton spicule)–the most upregulated was LOC100889419, an uncharacterized gene that maps most closely to the mucin family in lancelets, proteins generally included in mucous secretions. Also upregulated was mmp24, a matrix metallopeptidase. Matrix metallopeptidases help degrade the extracellular matrix allowing for mesenchyme cell activity and may also bind to integrin αVβ3. Peroxidasin was also upregulated, and is known in other animals to play a role in both TH synthesis (9) and providing collagen-like structure to the extracellular matrix and calcite skeleton (64–66). Other proteins with known functions we found to be upregulated are carbonic anhydrase 2 and calumenin, essential components of calcite skeleton deposition, and msp130, a spicule matrix protein associated with early spicule formation.

#### 3.4.2 Thyroid Hormone Signaling

A great number of TH synthesis, transport, and signaling-related genes were regulated by T3 and T4 (**Figure 7**). Three potential TH transport genes, including SLC26A7, SLC16A2 (also known as Monocarboxylate transporter 8 or MCT8), and SLC5A5 (NIS-like; LOC584046) were dramatically upregulated by T4, principally in the oldest larvae examined (27d). SLC26A7 and SLC5A5 are transport iodide and SLC16A2 to transport TH in chordates (67–69).

The sea urchin homolog of the nuclear TH receptor was also upregulated by T4 exposure in older larvae, as well as two downstream signaling cascade proteins PKC alpha and PI3K.

Three iodothyronine deiodinases (responsible for transformation of T4 to T3 and eventual degradation of THs) were dramatically upregulated by TH exposure in older larvae (23d and 27d). One iodothyronine deiodinase (LOC577015) was one of the few genes upregulated strongly in gastrulae.

Peroxidasin, for the sea urchin thyroid peroxidase ortholog (an essential component of vertebrate TH synthesis), was upregulated. Cholinesterases which we hypothesized might be a source of tyrosine for TH synthesis (>40% thyroglobulin identity and higher tyrosine content) were mainly downregulated, with the exception of LOC100893063.

Sulfotransferases, responsible for the catalysis of TH to their sulfated analogues, were mainly downregulated in the oldest larvae (27d) but specific cytosolic sulfotransferases were upregulated in 23 day old larvae (LOC577458, LOC100889865, LOC589239, LOC584514).

#### 3.4.3 Nuclear Hormone Receptors

Some nuclear receptors, primarily in the NR1 family, were upregulated by THs (**Figure 8**). This included THRb (LOC584535), the TH receptor beta ortholog, as well as retinoic acid receptor alpha and beta (RAR; LOC574567 and LOC100887874). Most upregulated of the nuclear receptors was a putative ortholog of liver receptor homolog 1 (LRH-1; LOC587072). Intriguingly, one strongly upregulated nuclear receptor has no firm ortholog in chordates but could be placed in the NR1 family (LOC756187) and showed some similarity to Rev-Erb—other orthologues of which were also upregulated (LOC578618, LOC115917889, and LOC580686). Notably, we did not find that the retinoid X receptor (RXR) was differentially regulated by THs.

#### 3.4.4 Apoptosis

56 apoptosis-related genes were regulated by T4 in older larvae (**Figure 9**). The most highly upregulated and downregulated apoptosis-related genes are putative orthologs of cytochrome C, an activator of apoptosis (LOC575347 and LOC582864 respectively). Other anti-apoptotic genes and cell growth promotors are upregulated, including mxd1, Six1, trim71, and kif11 and GRK5 (LOC577815, LOC587141, Six1, LOC579482, LOC373241, LOC756375). Six1 and nfia (LOC105437035) were also upregulated in gastrulae exposed to T4.

In contrast, several activators of apoptosis are also upregulated, particularly in post-rudiment larvae (27d), including Sox17-like, ppp1c, STK17A, and E2F (LOC587141, LOC752338, LOC582485, E2E3). Two important proapoptotic genes are also suppressed, CEBPA and tp63 (LOC 100893285 and LOC756859).

We did not find any caspases to be statistically significant DEGs, although the effector caspase, caspase 3/7 (LOC587820) did trend to downregulation (1.31-fold) by T4 in 27 day-old larvae, but not 23 day-old larvae.

#### 3.4.5 Adhesome/Cadhesome

T4 significantly upregulated cadhesome and adhesome-related genes, while downregulating two tight junction-related genes (**Figure 10**). Of the integrin adhesome genes, integrin alpha-PS1 was upregulated in 27 day old larvae, while integrin alpha-8-like was severely downregulated (LOC584174 and LOC105436778). We found no significant effect of THs on the expression of other integrins. Other adhesome genes involved in extracellular matrix secretion, adhesion, and regulation were upregulated, including dnajb1, PPIB, filamin, tensin1, and Sorbs1 (LOC574994, LOC577919, LOC587438, LOC115919752, LOC574636). In contrast, two orthologs of tgm1, an enzyme which crosslinks proteins increasing stability of the ECM, were downregulated (LOC592750).

Four cadherin orthologs (LOC100891456, LOC580257, LOC105442843, and LOC580453) were significantly upregulated by T4 in 27 day old larvae. As well, two orthologues of Rap1A (responsible for regulating cadherin-mediated cell adhesion; LOC115919354, LOC580151), TRPC4 (a Ca^2+^ channel which interacts with Cadherin to regulate cell adhesion; LOC586872), ARHGAP21 (enhances cell adhesion and necessary for epithelial-to-mesenchyme transition; LOC115918380), Shroom3 (interacts with cadherin and actin to control morphology; LOC578304), Delta-2-catenin (complexes with cadherins and actin, LOC594353). A cadherin/flamingo ortholog (LOC100891456) was a rare example of a gene upregulated by T4 in gastrulae.

Other structural and regulatory proteins associated with both cadherin and integrin cell adhesion were upregulated, including two filamins (LOC587438 and LOC479510), pard3 (LOC580534), and MAGUK (LOC752750). The two essential tight junction genes tetraspanin and melanotransferrin were both significantly downregulated by T4 in 23 day- and 27 day-old larvae (LOC751836 and LOC581063).

#### 3.4.6 Neuronal signaling

While most neuronal genes we examined were not regulated by T4 (**Supplement S1**, **S2**), the DEGs we found showed dramatic increases and decreases in expression (**Figure 11**). Most neural peptide precursors we examined showed little change in expression, however the thyrotropin-releasing hormone precursor Trh (LOC580381) was upregulated by T4 in 27 day old larvae.

Notably, the dopamine and serotonin synthesis enzyme, AADC, was upregulated by T4 in both 23d and 27d groups (LOC593847), while the serotonin synthesis enzyme tryptophan hydroxylase (LOC581035) and two serotonin receptor orthologs LOC581142 and LOC574784 were downregulated.

The GABA receptor GABRG2 (LOC762549) was upregulated in gastrulae, but not strongly regulated in older larvae. However, the two orthologs of the GABA transporter S6A13 (LOC585204 and LOC764578) were downregulated in older larvae (23d and 27d).

The acetylcholine receptor genes nAChRa7 (LOC764011) and muscarinic M4R (LOC115919284) were upregulated in both 23d and 27d groups, but not detectable in gastrulae. In contrast, Muscarinic M2RR and nAChRa9 (LOC592935, LOC582635) were downregulated in both older groups (23d and 27d).

Histamine N-methyltransferase orthologs, responsible for the methylation and metabolism of histamine were regulated by T4 in 23 day-old larvae, but less so in the older 27 day-old larvae. In contrast, the histamine H2 and H1 receptors were highly upregulated by T4 in 27 day-old larvae, but less so in 23 day-old larvae. As a general trend, histamine degradation genes were more pronounced in 23 day-old larvae while histamine receptor expression was more enhanced by T4 in 27 day-old larvae.

The noradrenaline transporter SLC6A2 (LOC105445726) is notable for being upregulated by T4 in gastrulae and 23 day-old larvae, but not in 27 day-old larvae. A sea urchin ortholog of the beta-1 adrenergic receptor (LOC577816), which binds to adrenaline and noradrenaline, was upregulated by T4 in 23d and 27d larvae. No other adrenergic system-related genes were found to be regulated by T3 or T4.

Of genes regulating neural development and differentiation, zic and emx (promotors of neurogenesis; LOC578589, LOC577702) were upregulated in older larvae, while SoxB1 (repressor of neurogenesis; SoxB1) was downregulated.

#### 3.4.7 Immune genes

We found fewer immune-related DEGs compared to the other categories annotated (**Figure 12**). Several immune-related genes were differentially upregulated by T3 and T4, including MDA5, (a viral recognition receptor; LOC115921168), LOC754218, (a putative ortholog of MIF, which regulates the innate immune system and also plays a role in integrin-mediated MAPK signaling and adhesion), and several SRCR domain-containing genes which may mediate innate immune response at the cell membrane (LOC100888472, LOC100889653, LOC105441964, LOC105447538, LOC578206, LOC764257), as well as an alpha-GalNAc lectin (LOC585907).

Also upregulated by T4 is an IRF ortholog (LOC115918232). Pias2 and SOCS2, repressors of the JAK/STAT pathway, are both also upregulated by T4 in 23 day- and 27-day old larvae (LOC578583, LOC594503). Several proteases/cytolytic genes are also upregulated (LOC105438173, LOC577926) or downregulated (LOC763692, LOC593009) by T4 in older larvae.

Plasminogen, a clotting precursor, is a rare example of a gene upregulated by T3 but downregulated by T4 (LOC105444510). Two other sea urchin-specific clotting genes (amassins; AMAS2, AMAS3) which enhance coelomocyte adhesion, are also downregulated by T4 in older larvae.

### 3.5 Thyroid hormone response element enrichment near differentially regulated genes

**Figure 13:**
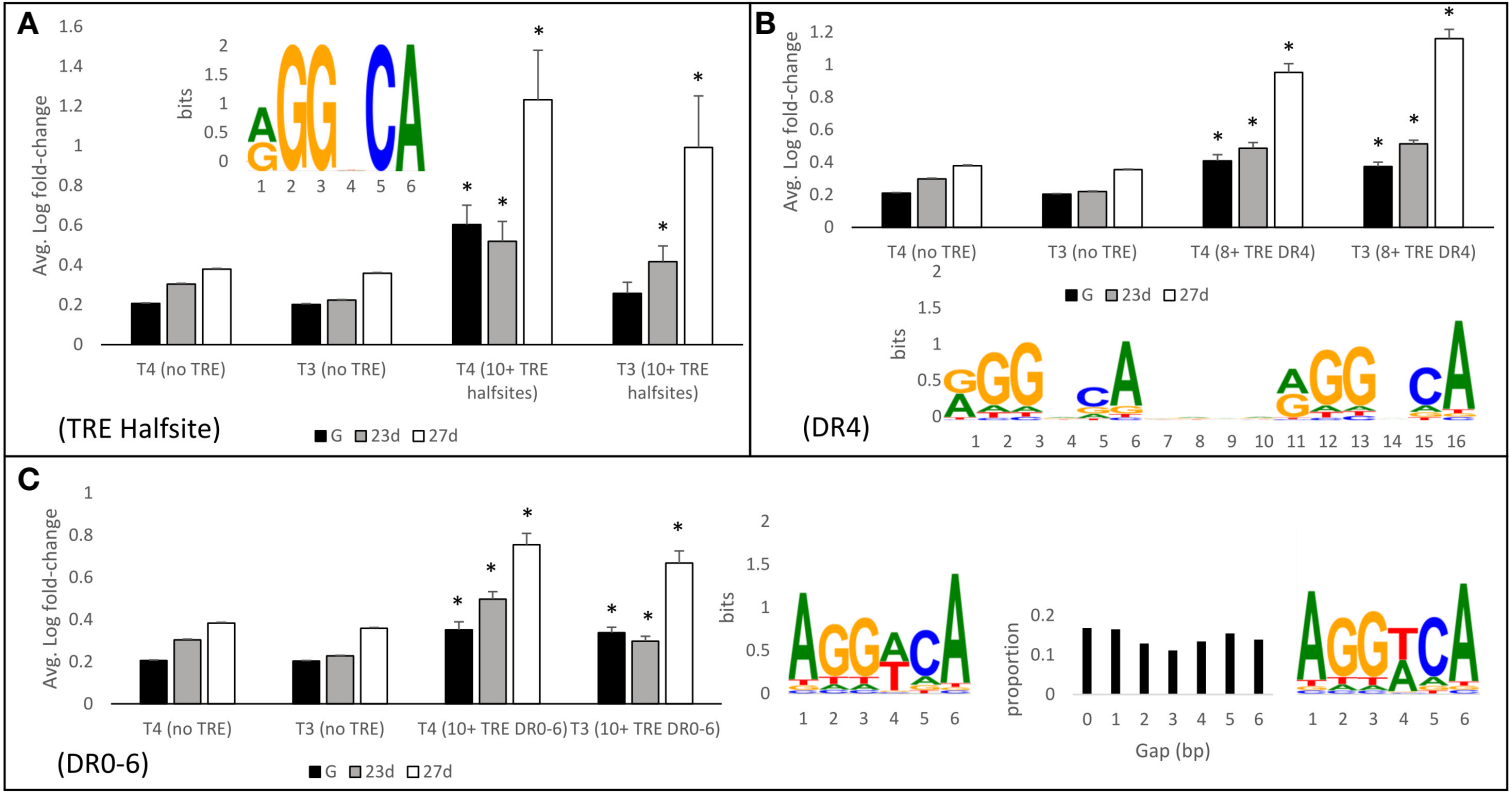
Genes with thyroid hormone response elements (TREs) <500 bp upstream are more likely to be regulated by THs. **(A)** Genes with >10 nearby TRE halfsites were regulated an average of 2.6-fold and 2.0-fold more strongly by T4 and T3 respectively when compared with genes that have no nearby TRE halfsites. Similarly, genes with nearby DR4 **(B)** and DR0-6 **(C)** sites were more heavily regulated by THs than genes with no nearby TRE sites. Nearby TRE sites were the strongest predictor of regulation in 27-day-old larvae, compared to younger larvae and gastrulae. * represents p<0.05 by two tailed T test comparison to sites with no nearby TRE.

Genes with nearby thyroid hormone response elements (TREs) were more likely to be regulated by THs (**Figure 13**). The subset of the transcriptome with a large number of TREs (>10) within 500 bp upstream or 20 bp downstream of the predicted transcription initiation site were significantly more likely to be regulated by T4 and T3. For example, genes with greater than 10 nearby TRE halfsites were regulated 2.6-fold and 2.0-fold more strongly by T4 and T3 respectively in 27 day-old larvae when compared with genes that have no nearby TRE halfsites (t-test, p<0.05). Similarly, gene initiation sites with nearby DR4 TREs (the canonical TH response element motif), were 1.8-fold and 2.2-fold regulated by T4 and T3 respectively, while genes with a more permissive search pattern allowing for DR0-6 motifs were 1.7-fold and 1.6-fold upregulated by T4 and T3 (t-test, p<0.05).

In all cases, regulation of genes with nearby TREs was highest in 27 day-old larvae, and lower in 23 day-old larvae and in gastrulae. Generally, gastrulae showed the lowest degree of gene regulation of genes with nearby TREs by T4/T3, however there was still a significant increase in gene regulation in gastrulae by T4 with all examined motifs, and by T3 with DR4 and DR0-6 motifs.

**Figure 14:**
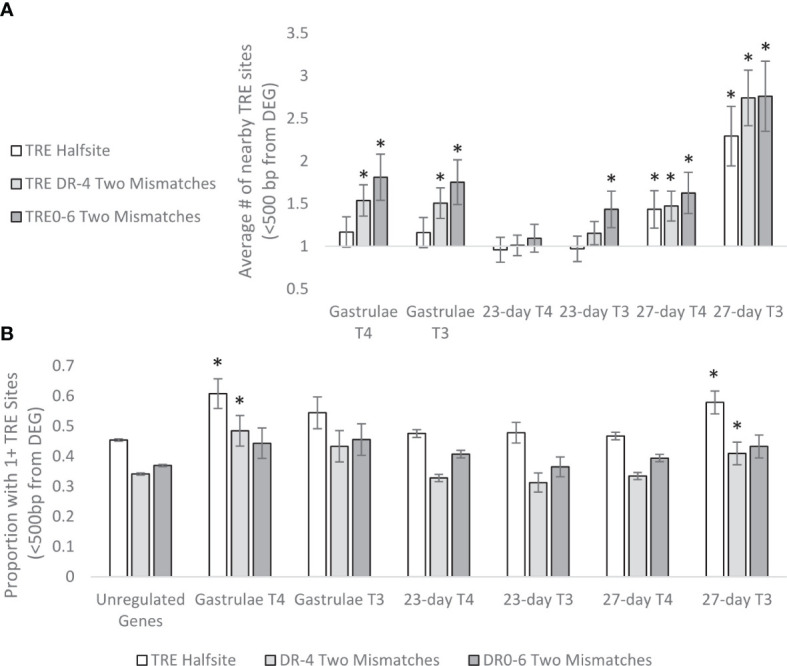
**(A)** Average enrichment of predicted TRE sites within <500 bp upstream of >2-fold DEG transcription initiation sites, relative to unregulated genes. Genes which are at least 2-fold upregulated by THs are more likely to have nearby (<500bp) TRE sites. In 23 and 27-day old larvae, T3 requires more nearby TRE sites to regulate genes than T4. In particular, genes sensitive to T3 regulation in 27-day old larvae had over 2-fold enrichment of nearby TRE halfsites, DR4 and DR0-6 predicted sites. * represents p<0.05 by two tailed T test comparison to unregulated genes. **(B)** Proportion of DEGs with nearby predicted TRE sites. Significantly more TRE halfsites and DR4 sites were present in genes regulated by T4 in Gastrulae, and T3 in 27-day-old larvae, with a trend of increased TRE sites near DEGs in 23-day-old larvae as well. * represents p<0.05 by two-tailed T test comparison to unregulated genes.

We also examined the number of TRE sites within proximity of detected DEGs and found a similar pattern (**Figure 14**). DEGs typically had more nearby TRE sites compared to non-regulated genes, with a peak of 2.3-fold more TRE halfsites, 2.7-fold more DR4 sites, and 2.76-fold more DR0-6 sites nearby genes differentially regulated by T3 in 27 day-old larvae. Generally, genes regulated by T3 and genes regulated in gastrulae required more nearby TRE sites when compared to genes regulated by T4 in 27-day old larvae (t-test, p<0.05).

## 4 Discussion

### 4.1 Evidence for thyroid hormone binding and signaling in sea urchin development

#### 4.1.1 Evidence for nongenomic signaling

We found that THs bound to membrane protein extract from *S. purpuratus* gastrulae. The binding constant of T4 is within 95% CI of that detected in a mammals (CV-1 cell line; 70). However, we found a greater affinity of T3 in sea urchins compared to mammals but still magnitudes lower than T4. Lower still was the affinity of Tetrac and T2, while we were not able to find binding of rT3 or Triac. The ability of RGD peptide to displace fluorescently conjugated T4 provides further evidence that T4 binds integrin near or within the RGD-binding pocket, an evolutionarily ancient region on integrins (71, 72). While T4 has a higher affinity to the integrin receptor and greater physiological effect, previous work has shown that T3 is present at roughly 50-fold higher concentration than T4. Both THs may have physiological relevance in sea urchins (3). It should be noted that up to 10% cytosolic proteins may remain in a membrane protein fraction (73). However, fluorescently labeled thyroid hormones were displaced by an integrin ligand, RGD peptide, which is not known to bind to the nuclear membrane receptor, and THs have been found previously not to bind to cytosolic proteins in sea urchins (74), increasing the likelihood that our binding constants reflect binding to an integrin membrane protein.

Fewer DEGs were detected in gastrulae as compared to older larvae. The DEGs that were found were less tightly linked to TRE presence and less likely to show a greater magnitude of differential expression when exposed to T4 compared to T3. Most notable is the upregulation of iodothyronine deiodinase by both T3 and T4 in gastrulae. This suggests that TH metabolism may be active early in development. We consider this to be likely, especially given the high concentration of iodinated tyrosine derivatives in a typical larval diet (3, 75) and the potential for larvae to derive THs from them (Vitamin hypothesis; 1).

When it comes to T3, the ratio of upregulated to downregulated DEGs was lower than 50%, which is universally not the case in TH exposure transcriptomes (E.g. 18, 21, 22). As well, the total number of detected DEGs was much lower than in T4-exposed groups. These data suggest that T3, if acting via the nuclear hormone receptor, does so to a lesser degree than T4 and that the nongenomic effects of T3 may be more prominent. Future work should test the effects of RGD and PD98059 inhibition on T3 acceleration of skeletogenesis to determine whether it is affected to a lesser or greater degree than T4, and testing whether T3 acts primarily via the membrane receptor in sea urchins. This would shed light on the relative importance of T3 versus T4 binding.

#### 4.1.2 Evidence for genomic signaling

Previous work has shown that in gastrulae, the effect of THs on skeletogenesis was completely inhibited by high levels of RGD peptide, a competitive ligand of the integrin membrane receptor to which T4 potentially binds (13). As well, inhibition of MAPK ERK1/2 prevented the acceleration of skeletogenesis by T4 in gastrulae (13). While this previous work also showed T4-induced acceleration of skeletogenesis in the rudiment, it did not test the mechanism involved. We now show that during development of the sea urchin rudiment, acceleration of skeletogenesis by T4 is potentially decreased by RGD peptide and PD98059, but not completely suppressed. These data suggest a role for T4 regulation of metamorphic development by genomic means as well.

Genomic regulation by THs via the nuclear TH receptor is characterized in chordates by the regulation of a large number of genes (primarily upregulation) over a period of hours to days. When we exposed sea urchin gastrulae and larvae to THs for a period of 24 hours, we saw some regulation of gene expression in gastrulae, but many more genes regulated in older larvae, both pre- and post- initiation of rudiment development. This provides further evidence that while T4 may accelerate skeletogenesis in gastrulae primarily via nongenomic means, T4 regulation of gene expression by genomic means is a major force in larval development to metamorphosis.

However, the nuclear TH receptor is expressed in gastrulae as well and TRE analysis revealed some genes responsive to T4 are also more highly associated with putative nuclear TH receptor binding sites on the genome. Genes regulated by THs are known to have enriched TRE presence (61). That we find a significant association of TRE sites with TH-induced DEGs in sea urchin larvae provides strong evidence for genomic signaling activity.

Cocurullo et al. (76) performed a single cell transcriptomic analysis of nuclear thyroid hormone receptor expression, suggesting that THR is expressed in sea urchin gastrulae in skeletogenic cells, globular cells, oral ectoderm, and anterior neuroectoderm, as well as in the coelomic pouch and muscle cells of pluteus larvae. The authors confirmed strong expression of THR in skeletogenic mesenchyme cells with fluorescence *in-situ* hybridization (FISH). This suggests that action via THR may also play a role during early larval development.

Genomic TH signaling may play a significant part in regulation of sea urchin development. The stronger association of DEGs with TREs in post-rudiment development larvae suggests an association of genomic signaling with later stages of development, especially development to metamorphosis. In post-rudiment larvae, the nuclear THR was upregulated by T4, indicating autoinduction of THR signaling, a classic signature of TH signaling via the nuclear hormone receptor during metamorphosis (77, 78). As THR levels are not significantly different between older larvae and gastrulae, the increased autoinduction in older larvae may partially explain the greater number of DEGs. We propose that while THs accelerate skeletogenesis primarily via non-genomic means in gastrulae, they may regulate other physiological systems—especially in older larvae—via a genomic mechanism.

#### 4.1.3 Differential regulation by genomic and nongenomic pathways

n our analysis, we frequently found that TH effects in gastrulae were opposite in direction to those in 23 day-old and 27 day-old larvae. We have proposed that nongenomic signaling may be more prominent during gastrulation, and so a potential explanation for the contrasting effects of THs in gastrulae and pluteus larvae is differential regulation depending on the pathway.

THs are known to have differential effects via non-genomic and genomic pathways in chordates. These effects can be antagonistic or synergistic. Wang et al. (79) knocked down the nuclear TH receptor in frog tadpoles and found that T3 still regulated gene expression. The authors provided evidence that the immune and metabolic effects of T3 in chordates may depend on nongenomic signaling and found differential effects of the genomic and nongenomic pathways on apoptosis and regulation of development. Notably, Wang et al. found that 33% of total DEGs were regulated by T3 in TR-knockdown tadpoles, with only 8% of TR+ DEGs being regulated in the same fashion in TR-knockdown tadpoles.

It is intriguing that the most enriched GOslim categories in gastrulae on exposure to T4 were microtubule binding and cytoskeletal protein binding (MF) and regulation of microtubule-based process (BP). This is consistent with T4 regulation of primary mesenchyme cells in gastrulae via an integrin membrane receptor. THs are well-known to regulate actin processes in chordate (14, 80), which is closely associated with mesenchyme cell regulation and the epithelial-to-mesenchyme transition (81). In contrast, late-stage pluteus larvae showed greater regulation by THs of apoptosis, immune, neuronal, and signaling-related processes. These differences may be accounted for by the specific TH signaling pathways active during each developmental stage, and by the cell types expressing integrin membrane receptors and nuclear TH receptors.

#### 4.1.4 Sulfated THs

In-silico docking models have suggested that sulfated THs may bind with high affinity to the integrin membrane receptor, integrin αvβ3 (82). Low levels of sulfated THs have previously been detected in chordate neural tissue (83). Consequently, we included sulfotransferases in our analysis of TH metabolism. We found four cytosolic sulfotransferases which were upregulated by T4 in larvae with no juvenile rudiment (23d), but six sulfotransferases to be downregulated by T4 in larvae which had begun rudiment development (27d). This suggests that there may be a differential sulfation of THs pre- and post- rudiment development. Sulfation of THs is also an essential step in catabolism and degradation (84–86). Additionally, iodothyronine deiodinase orthologs were highly upregulated. Iodothyronine deiodinases preferentially target sulfated TH derivatives, which are degraded more quickly than T4 (87, 88). Therefore, our analysis suggests that TH sulfation may be attenuated by T4 exposure in larvae with developing rudiments. In contrast, sulfotransferases are either unregulated or slightly upregulated in gastrulae, and iodothyronine deiodinases are not dramatically upregulated, suggesting a potentially greater presence of sulfated TH derivatives in gastrulae.

#### 4.1.5 Thyronamines

A thyronamine, 3T1AM, has been shown to cause nongenomic effects in chordates (89). 3T1AM may be derived from T4 or T3, although the biosynthesis pathways are unclear (90) and the physiological relevance of thyronamines is still unknown (91). Ornithine decarboxylase has been proposed as the enzyme responsible for the decarboxylation of THs into biologically active thyronamines partially responsible for the nongenomic actions of T4, and the expression has been found to be regulated in hypothyroid mice, but not by T4 exposure (92). Similarly, we found no regulation of ornithine decarboxylase by T3 or T4 in our transcriptomic analysis. The alpha-2A adrenergic receptor (Adra2a) has previously been suggested as a receptor for 3T1AM (93, 94). We did find significant upregulation of Adra2a in 23d and 27d groups, but did not find regulation of any other proposed aminated T4 receptors or transporters. If Adra2a is a physiologically relevant 3T1AM receptor in sea urchins, we have provided evidence of positive feedback driven by T4-exposure.

### 4.2 Thyroid hormone-regulated skeletogenesis in sea urchin development

Of skeletogenesis-related genes significantly regulated in the TH-exposed groups, 100% were upregulated in gastrulae, and 56% in late-stage larvae. However, the magnitude of the regulation was lower in gastrulae, with critical skeletogenesis-related genes not detected as significantly different in the TH-exposed groups. This is at-odds with the physiological effects caused by THs in early gastrulation in *S. purpuratus* (and other echinoderms, including *Dendraster excentricus* and *Ophiopholis aculeata*; 95 [in preparation]). We hypothesize that much of the regulation of skeletogenesis in early stages may act via the integrin receptor, non-genomically, whereas the nuclear TH receptor plays a more active role during later stages, especially during rudiment formation and metamorphosis. Phosphorylation cascades triggered by the integrin membrane receptor may allow for rapid regulation of skeleton deposition over short time periods not suitable for transcriptional regulation by the nuclear thyroid hormone receptor (<5 hours; 13).

We detected differences between the gastrula stage and later stages with respect to TH effects, despite evidence that the skeletogenic gene regulatory networks are similar (96). In gastrulae, the upregulated skeletogenesis-related genes are effector genes, either from the spicule proteome or known to be expressed in PMCs, e.g. SM-30, and MSP130, two of the most abundant and important spicule matrix proteins. In late-stage larvae, the upregulated genes are related to both skeletogenesis and other developmental processes such as the remodeling of extracellular matrix and the epithelial-mesenchyme transition more generally. However, most spicule proteome genes are still upregulated in late-stage, including the sm-29, sm30 families, and MSP130. Juvenile skeleton comprises multiple complex elements, including juvenile spines, tube feet, and test, which develop under spatial and temporal regulation (39). It is possible that genomic TH regulation of skeletogenesis during rudiment development allows for regulation of a more complex process, relative to embryonic skeletogenesis. While the known juvenile gene regulatory network (GRN) governing skeletogenesis in the juvenile rudiment shares features with the gastrula GRN, there are also significant differences, including entirely missing signaling modules (PMAR-HesC Tbrain, Tel, FoxB/FoxO; 96). One possibility is that TH signaling and other signaling mechanisms controlling development to metamorphosis may replace missing regulatory modules, allowing for mesenchyme cell specification and regulation.

In previous work, we found that a 90-minute exposure to THs increased expression of Ets1, a transcription factor regulating skeletogenesis, in gastrulae. In this 24-hour exposure we find no significant effect on Ets1 levels. However, we have found upregulation of genes directly downstream of Ets1. We expect that Ets1 upregulation may be a stage- cell-specific result of MAPK ERK1/2 signaling induced by T4 exposure. By the end of gastrulation, Ets1 is no longer expressed in primary mesenchyme cells, and may therefore not be accessible to regulation by THs (97). We do not see higher expression of VEGF in the T4-exposed groups. VEGF is typically believed to be the primary external signaling factor responsible for skeletogenesis in sea urchin primary mesenchyme cells. Given that both the integrin membrane receptor and nuclear hormone receptor are expressed by the primary mesenchyme cells, we propose that a direct regulation of PMCs by THs is more likely than a VEGF-mediated mechanism.

### 4.3 Regulation of other functional groups by thyroid hormones

#### 4.3.1 Programmed cell death (PCD)

PCD is a critical process in both chordate and non-chordate metamorphosis (reviewed in 98). Apoptosis in particular plays an important role in morphogenesis during sea urchin larval development (38, 99–101). We previously found that THs regulate larval arm retraction via programmed cell death in sea urchin larvae, with increased levels of caspase 3/7 activation and apoptosis (38). In this transcriptome analysis, we find that caspase gene expression was not affected by THs.

We found increased transcription of activators of apoptosis, including Sox17, protein phosphatase 1 catalytic subunit (ppp1c), STK17A, and E2F. These proteins have been previously described increasing apoptosis and caspase 3/7 activation in chordates (102–105). SOX17 in particular is associated with activation of the intrinsic pathway and caspase 3/7 cleavage (104). Crucially, the most upregulated apoptosis-related gene is a variant of cytochrome C (LOC575347). Cytochrome C is responsible for binding apaf-1 and procaspase-9 leading to the formation of the apoptosome and subsequent cleavage/activation of caspase-9 and activation of the intrinsic pathway (98). Therefore, we propose that THs may increase caspase activation without increasing caspase transcription.

#### 4.3.2 Adhesome (Cadhesome/Septate Junction) proteins

While genes involved in extracellular matrix secretion, adhesion, and regulation were upregulated, two orthologs of tgm1, an enzyme known to increase ECM stability, were downregulated. These data suggest that THs may increase ECM deposition and remodeling in older larvae. Additionally, cadhesome-related genes were generally upregulated, with the exception of fascin orthologs. Four cadherins were upregulated by T4, including an ortholog of N-Cadherin, a cadherin associated with mesenchyme cells and the epithelial to mesenchyme transition (106, 107). A switch of expressed cadherin types is thought to be essential for the epithelial to mesenchyme cell transition (108), therefore changes in cadherin expression may allow for cell motility and tissue remodeling during development. A decrease in Cadherin-E expression regulated by Snail is necessary for EMT during sea urchin gastrulation (109–112). Snail is also upregulated in T4-exposed older larvae, and is downstream of Ets1, a gene we propose to be regulated by T4 via nongenomic MAPK cascade (13). Evidence suggests Snail may participate in T4 regulation of cadherin expression.

Tight junction-related genes are typically downregulated during EMT, a process which has also been linked to Snail expression (113). Sea urchin septate junction genes share similarities with chordate tight junctions (114). Tetraspanin and melanotransferrin, two essential tight junction/septate junction genes, were both significantly downregulated by T4 in older larvae. Tetraspanins are often inhibitors of the epithelial to mesenchyme transition, and the closest vertebrate ortholog to the most abundant sea urchin tetraspanin, tetraspanin18, also acts to inhibit EMT (115). We did not find significant regulation of Mesh, an integral component of gut septate junction in pluteus larvae, (114), although several orthologs trended to downregulation by T4 in post-rudiment larvae (LOC580458, LOC574757, LOC105439366). Taken together, these data suggest T4 regulation in pre- and post-metamorphic sea urchin plutei increases cell motility, facilitates the epithelial to mesenchyme transition, and enhances tissue remodeling prior to metamorphosis.

#### 4.3.3 Neuronal signaling and crosstalk

76 found expression of the nuclear TH receptor in neuronal cells of 3-day old pluteus larvae using a single cell transcriptomics approach and the authors suggest that THs may colocalize with serotonergic neurons described as “Sp-Pdx1/Sp-Brn1/2/4 expressing neurons”. We find in our transcriptome analysis that THs induce a strong upregulation of Aromatic L-amino acid decarboxylase (AADC), a key serotonin (5-HT) synthesis enzyme, as well as downregulation of two serotonin receptors. This coincides with previous research on chordates: THs have previously been found to decrease serotonin receptor levels and increase serotonin levels in chordates (116, 117). The nuclear TH receptor in sea urchins colocalizes to neurons expressing the sea urchin serotonin receptor (118). We found T4 downregulated two serotonin receptors, one of which (LOC581142) was found to colocalize with the nuclear TH receptor by Paganos et al. (118). Paganos et al. (118) also describe several glucose co-transporter genes to be markers of this cell type, which we found to be downregulated by T4 in older larvae (Slc5a9 and Slc2a1). Together, these data suggest regulation of “Sp-Pdx1/Sp-Brn1/2/4 expressing neurons” by THs, reducing sensitivity of these neurons to both serotonin and glucose. In chordates, THs regulate and induce differentiation of Pdx1-expressing cells (119). While THs slightly increased Pdx1 and Brn1/2/4 levels in older larvae, it was not to a significant extent, and we found no support for the hypothesis that THs control differentiation of these neurons during the developmental stages we tested.

A serotonergic nervous system has also been described in older pluteus larvae, with axons reaching from the apical ganglion to the larval arms and juvenile rudiment (120). In this context, nuclear TR colocalization with and regulation of serotonergic neurons has implications for TH control of juvenile rudiment development and metamorphosis. Excision of the serotonergic neurons along with the pre-oral hood, resulted in spontaneous metamorphosis of sea urchin larvae, while electrical stimulation of these neurons resulted in a greater degree of metamorphosis (121). The downregulation of serotonergic receptors we observed might therefore be one of the mechanisms by which THs stimulate metamorphosis.

Previous research has shown that THs induce apoptosis in sea urchins (38) and that the histaminergic system may be crucial for regulation of metamorphosis and settlement, with histamine accelerating metamorphic competence but inhibiting settlement and apoptosis in the larval arms (52, 122). We find that in post-rudiment larvae (27d), T4 significantly increased histamine receptor expression, while in pre-rudiment larvae (23d), T4 increased expression of histamine-metabolizing enzymes. Histamine crosstalk may play a role in TH acceleration of metamorphic competence, with T4 increasing histamine receptor expression to accelerate rudiment development. This provides further evidence that crosstalk between these two signaling pathways is important for regulation of development to metamorphosis in sea urchins, as originally proposed in Sutherby et al. (122).

The adrenergic system has been linked with nongenomic TH signaling in chordates, and THs have been proposed as a neurotransmitter (27, 29, 123). We found that T4 (and to a lesser degree T3) upregulated beta-1 adrenergic receptor and the noradrenaline transporter SLC6A2 orthologs in our transcriptome analysis. While little work has been done on sea urchin adrenergic receptors, adrenergic signaling has been shown to be necessary for tube foot motility in adult sea urchins (124).

Retinoic acid signaling has been implicated in metamorphosis of numerous non-sea urchin echinoderms (125) and can cause pseudopodial cable growth in sea urchin skeletogenic mesenchyme cells in culture (126). We found that thyroid hormones strongly upregulated both orthologs of the retinoic acid receptor, suggesting a possible link between thyroid hormone exposure and retinoic acid receptivity.

#### 4.3.4 Innate immune system

Echinoderms possess an innate immune system with a diverse array of immune recognition receptors (57, 127). THs can regulate immune response in chordates (16), and remodeling of the immune system is implicated in chordate metamorphosis (128, 129). We found that some immune-related genes were highly upregulated by T4, including a variety of SRCR-domain containing proteins, a protein family which has been greatly expanded in echinoderms and is involved in innate immune response (130–133). A few immune DEGs were also regulated in gastrulae, suggesting a potential non-genomic response to THs. However, we found fewer immune-related DEGs than the other categories of genes we manually annotated.

### 4.4 Evolution of thyroid hormone regulation of development

TH effects have long been described in non-chordates, but until recently, the mechanisms were not understood (reviewed in 1, 2). Non-chordates do not possess a thyroid gland but are capable of TH synthesis and receptor function (2, 134). Orthologs of all necessary TH signaling system components are present in Bilateria, with TH synthesis having been described even in prokaryotes (2).

Previously, the TH T4 has been described binding to nuclear extract from a sea urchin larva (74). We have provided evidence linking TH action to genomic signaling via the nuclear hormone receptor. Our data suggest that actions of THs on older sea urchin larvae can be attributed at least in part to genomic signaling via a nuclear hormone receptor, likely THRb. Along with previous evidence linking THRb to regulation of metamorphosis by THs in molluscs and annelids (62, 135) and the fact the THR signaling is likely a bilaterian innovation (37), it is plausible that TH signaling is widespread among bilaterians.

Nongenomic signaling has not yet been demonstrated in a non-deuterostome, having only been shown in sea urchins and vertebrates. However, the proteins and domains essential for non-genomic signaling are also evolutionarily ancient (37). Future research should explore this integrin mediated non-genomic TH signaling pathway in these groups.

## 5 Conclusion

Our data provide further evidence that TH signaling can occur *via* an integrin membrane receptor in sea urchin development and that both genomic and non-genomic signals are involved in the regulation of skeletogenesis in sea urchins. Our detailed transcriptional analysis suggests that TH signaling in later stages of development involves the nuclear TH receptor in addition to non-genomic actions. Furthermore, differentially expressed genes in response to THs cover a broad range of physiological and developmental processes, previously implicated in TH signaling in chordates, such as apoptosis, cell adhesion, neuronal signaling and morphogenesis. Together with previously published evidence that THs are synthesized by sea urchin embryos and larvae and the role of THs in metamorphic development, our data suggest that the function of THs in development is a shared feature of bilaterians or even animals.

## Data availability statement

The datasets presented in this study can be found in online repositories. The names of the repository/repositories and accession number(s) can be found below: https://www.ncbi.nlm.nih.gov/, PRJNA949545.

## Author contributions

ET and AH were involved in all experimental design. All authors designed the transcriptome hormone exposure. ET and HW performed the transcriptome hormone exposure and collected samples. ET performed the morphological assays and binding assays, conducted the statistical analyses, the transcriptome analysis, and the thyroid hormone response element analysis. ET wrote the first draft of the manuscript. All authors contributed to the article and approved the submitted version.
