# Supplementary material for: Thyroid hormone membrane receptor binding and transcriptional regulation in the sea urchin Strongylocentrotus purpuratus
Source: Front Endocrinol (Lausanne). 2023 May 26;14:1195733. doi: 10.3389/fendo.2023.1195733 (PMC10250714; doi:10.3389/fendo.2023.1195733)
Supplement: Supplementary file 4 [file DataSheet_4.zip › 27dpf_T3_rep2_16_S25_L008_XR1_001_val_1_fastqc.html]

27dpf\_T3\_rep2\_16\_S25\_L008\_X1\_001\_val\_1.fq.gz FastQC Report 

FastQC Report

Mon 14 Sep 2020  
27dpf\_T3\_rep2\_16\_S25\_L008\_X1\_001\_val\_1.fq.gz

## Summary

- Basic Statistics
- Per base sequence quality
- Per tile sequence quality
- Per sequence quality scores
- Per base sequence content
- Per sequence GC content
- Per base N content
- Sequence Length Distribution
- Sequence Duplication Levels
- Overrepresented sequences
- Adapter Content

## Basic Statistics

| Measure | Value |
| --- | --- |
| Filename | 27dpf\_T3\_rep2\_16\_S25\_L008\_X1\_001\_val\_1.fq.gz |
| File type | Conventional base calls |
| Encoding | Sanger / Illumina 1.9 |
| Total Sequences | 17106146 |
| Sequences flagged as poor quality | 0 |
| Sequence length | 36-126 |
| %GC | 40 |

## Per base sequence quality

## Per tile sequence quality

## Per sequence quality scores

## Per base sequence content

## Per sequence GC content

## Per base N content

## Sequence Length Distribution

## Sequence Duplication Levels

## Overrepresented sequences

| Sequence | Count | Percentage | Possible Source |
| --- | --- | --- | --- |
| GCTACCTTCGCACGGTCAAGATACCGCGGCCGTTTAACCTCTAGTCACTG | 46262 | 0.2704408111564113 | No Hit |
| GCTGCTTCTAGGCCTATTCCTTCGAGAATAGCTATTTTGGGGCTTGTTGT | 43550 | 0.2545868601846377 | No Hit |
| CCATGGTTAAAAGCGTATAAAACGCAGGTGAAGTTATCTTACTTATATTA | 31890 | 0.1864242243694167 | No Hit |
| CGCAAACCTTTTCGTCAATGTGAACTCTCAGAAAAGATAACGCTGTTATC | 29803 | 0.1742239309777901 | No Hit |
| CCCCAACCAAAGCTTTTGCTAAATAGTTTAAAACTTACTGTTGGATAGGT | 27602 | 0.16135721044354467 | No Hit |
| TTAAAAGCTTCTGCACCCTTAGGATGTCCCGATCCAACATCGAGGTCGCA | 27291 | 0.15953915043166356 | No Hit |
| GCCCCAACCAAAGCTTTTGCTAAATAGTTTAAAACTTACTGTTGGATAGG | 26315 | 0.1538335987545061 | No Hit |
| CTTAAAAGCTTCTGCACCCTTAGGATGTCCCGATCCAACATCGAGGTCGC | 23557 | 0.13771073858483376 | No Hit |
| CAAATGATTATGCTACCTTCGCACGGTCAAGATACCGCGGCCGTTTAACC | 23175 | 0.13547762307184796 | No Hit |
| GTTTAACCTCTAGTCACTGGGCAGGCAGGACTCCCCATGCTTTAAATTCG | 23146 | 0.13530809336012917 | No Hit |
| GCACCCTTAGGATGTCCCGATCCAACATCGAGGTCGCAAACCTTTTCGTC | 23114 | 0.13512102609202564 | No Hit |
| GGCTTGTTGTCTTTAACTATCAATGTTGGATTTTTCCATTGTGGGCTTTA | 22718 | 0.13280606864924455 | No Hit |
| CTTTTTTTCCACTATTGCAACAGTGGGGGTATCCTTCTAAAAAGGAGGGA | 22409 | 0.1309997003416199 | No Hit |
| GTGAAATTCAGGGCTATGAAAAAGAGACAGTTTAGTTCCGTCTTGCCATT | 21697 | 0.12683745362631652 | No Hit |
| GCTTGTTGTCTTTAACTATCAATGTTGGATTTTTCCATTGTGGGCTTTAA | 21491 | 0.1256332080879001 | No Hit |
| GTCCTTTCGTACTAAGAGAGACCTTAACGTAGATAGAAACTGACCTGGCT | 21134 | 0.12354623887812018 | No Hit |
| GTTAGTCACAAGGTAAGTGTGTTAAAATTTTAACTTCCGCTTAAAGCTCG | 20528 | 0.12000365248840972 | No Hit |
| GGGGTATCCTTCTAAAAAGGAGGGAAGATTAGCTCGCCCAGTTTCGGGAT | 20515 | 0.11992765641074266 | No Hit |
| GCGGTAACTTGTTTCTTTGATCACCTTAGTGGATCATTCTTTCATTTTGA | 20495 | 0.11981073936817796 | No Hit |
| CCCAACCAAAGCTTTTGCTAAATAGTTTAAAACTTACTGTTGGATAGGTA | 20079 | 0.11737886488283217 | No Hit |
| GTTTTATTTTTCTTATAGAGGCTTAGCTCAAACTAATTTTTTGGGAAACC | 18991 | 0.11101857776731242 | No Hit |
| CTTAGCTCAAACTAATTTTTTGGGAAACCAGCTATCTCTGGGCGCGGTTA | 18069 | 0.10562870210507967 | No Hit |
| GGCTGCTTCTAGGCCTATTCCTTCGAGAATAGCTATTTTGGGGCTTGTTG | 17324 | 0.10127354226954453 | No Hit |
| CCCTAACCTCTGCTTAAAAATTAAAAGATTCTTTTTATAATATTAAAGGA | 17211 | 0.10061296097905396 | No Hit |
| GTGAAGTTATCTTACTTATATTAACATTGTCTCTTCTGATTACAGAATGT | 17178 | 0.1004200478588222 | No Hit |

## Adapter Content

Produced by FastQC (version 0.11.9)
