# Supplementary material for: Thyroid hormone membrane receptor binding and transcriptional regulation in the sea urchin Strongylocentrotus purpuratus
Source: Front Endocrinol (Lausanne). 2023 May 26;14:1195733. doi: 10.3389/fendo.2023.1195733 (PMC10250714; doi:10.3389/fendo.2023.1195733)
Supplement: Supplementary file 4 [file DataSheet_4.zip › 27dpf_T4_rep2_17_S26_L008_XR1_001_val_1_fastqc.html]

27dpf\_T4\_rep2\_17\_S26\_L008\_X1\_001\_val\_1.fq.gz FastQC Report 

FastQC Report

Mon 14 Sep 2020  
27dpf\_T4\_rep2\_17\_S26\_L008\_X1\_001\_val\_1.fq.gz

## Summary

- Basic Statistics
- Per base sequence quality
- Per tile sequence quality
- Per sequence quality scores
- Per base sequence content
- Per sequence GC content
- Per base N content
- Sequence Length Distribution
- Sequence Duplication Levels
- Overrepresented sequences
- Adapter Content

## Basic Statistics

| Measure | Value |
| --- | --- |
| Filename | 27dpf\_T4\_rep2\_17\_S26\_L008\_X1\_001\_val\_1.fq.gz |
| File type | Conventional base calls |
| Encoding | Sanger / Illumina 1.9 |
| Total Sequences | 14594481 |
| Sequences flagged as poor quality | 0 |
| Sequence length | 36-126 |
| %GC | 40 |

## Per base sequence quality

## Per tile sequence quality

## Per sequence quality scores

## Per base sequence content

## Per sequence GC content

## Per base N content

## Sequence Length Distribution

## Sequence Duplication Levels

## Overrepresented sequences

| Sequence | Count | Percentage | Possible Source |
| --- | --- | --- | --- |
| GCTGCTTCTAGGCCTATTCCTTCGAGAATAGCTATTTTGGGGCTTGTTGT | 17039 | 0.11674961240485358 | No Hit |
| GCTACCTTCGCACGGTCAAGATACCGCGGCCGTTTAACCTCTAGTCACTG | 15642 | 0.10717750086488174 | No Hit |

## Adapter Content

Produced by FastQC (version 0.11.9)
