# Supplementary material for: Thyroid hormone membrane receptor binding and transcriptional regulation in the sea urchin Strongylocentrotus purpuratus
Source: Front Endocrinol (Lausanne). 2023 May 26;14:1195733. doi: 10.3389/fendo.2023.1195733 (PMC10250714; doi:10.3389/fendo.2023.1195733)
Supplement: Supplementary file 4 [file DataSheet_4.zip › gastrula_T3_rep1_1_S10_L008_XR2_001_val_2_fastqc.html]

gastrula\_T3\_rep1\_1\_S10\_L008\_X2\_001\_val\_2.fq.gz FastQC Report 

FastQC Report

Mon 14 Sep 2020  
gastrula\_T3\_rep1\_1\_S10\_L008\_X2\_001\_val\_2.fq.gz

## Summary

- Basic Statistics
- Per base sequence quality
- Per tile sequence quality
- Per sequence quality scores
- Per base sequence content
- Per sequence GC content
- Per base N content
- Sequence Length Distribution
- Sequence Duplication Levels
- Overrepresented sequences
- Adapter Content

## Basic Statistics

| Measure | Value |
| --- | --- |
| Filename | gastrula\_T3\_rep1\_1\_S10\_L008\_X2\_001\_val\_2.fq.gz |
| File type | Conventional base calls |
| Encoding | Sanger / Illumina 1.9 |
| Total Sequences | 15800383 |
| Sequences flagged as poor quality | 0 |
| Sequence length | 36-126 |
| %GC | 39 |

## Per base sequence quality

## Per tile sequence quality

## Per sequence quality scores

## Per base sequence content

## Per sequence GC content

## Per base N content

## Sequence Length Distribution

## Sequence Duplication Levels

## Overrepresented sequences

| Sequence | Count | Percentage | Possible Source |
| --- | --- | --- | --- |
| AGAAAATCCAAACAATAAATTTCTATAAAATAGAAAACACTCTTACTCTC | 37997 | 0.24048151237852905 | No Hit |
| GAAAATCCAAACAATAAATTTCTATAAAATAGAAAACACTCTTACTCTCT | 16058 | 0.1016304478188915 | No Hit |
| AAAACATTGAAACTAAAAATCCTATTTTTAAAGTCGTCTTCCAACTCAGG | 15858 | 0.1003646557175228 | No Hit |

## Adapter Content

Produced by FastQC (version 0.11.9)
